# Analysis of complete mitochondrial genome sequence of bar-tailed Treecreeper *certhia himalayana* (psittaciformes: Certhiidae)

**DOI:** 10.1080/23802359.2021.1875909

**Published:** 2021-02-12

**Authors:** Li Xie, Ming Li, Yubao Duan

**Affiliations:** a School of Preclinical Medicine, Chengdu University, Chengdu, China; b Key Laboratory for Forest Resources Conservation and Utilization in the Southwest Mountains of China, Ministry of Education, Southwest Forestry University, Kunming, China; c Sichuan Kelun Pharmaceutical Research Institute, Chengdu, China

**Keywords:** Mitogenome, gene arrangement, phylogeny, Bar-tailed Treecreeper

## Abstract

Bar-tailed Treecreeper *Certhia himalayana* usually lives in coniferous or mixed broadleaf-conifer forests, often crawling along the trunk. In this study, we first sequenced and described the complete mitochondrial genome and phylogeny of *C. himalayana*. The whole genome of *C. himalayana* was 16,852 bp in length, and contained 13 protein-coding genes, 22 transfer RNA genes, 2 ribosome RNA genes, and 1 non-coding control regions. The overall base composition of the mitochondrial DNA was 25.1% for A, 29.2% for T, 14.5% for C, 31.2% for G, with a GC content of 45.7%. A phylogenetic tree strongly supported that *C. himalayana* closely related with Family Troglodytidae by highly probability.

Bar-tailed Treecreeper (*Certhia Himalayana* Vigors, 1832) inhabits coniferous forest or mixed broadleaf-conifer forest at an altitude of 1000-3500 meters, occasionally occurs in the shrub to c.500 m (BirdLife International 2016). *C. himalayana* occurs in Central Asia and southwest of China (Yunnan, Sichuan and Shanxi provinces) (Harrap and Quinn 1996). This species has an extremely large range, and hence does not approach the thresholds for Vulnerable under the range size criterion (BirdLife International 2016). Molecular studies supported *C. himalayana* closely related with Sittidae and Troglodytidae by highly probability based on mitochondrial genes, nuclear genes, both nuclear and mitochondrial genes, or mitochondrial and morphological data (Jonsson and Fjeldsa 2006). Barker et al (2002) and Fregin et al (2012) also proved it used nucleotide sequence or mitochondrial and nuclear genes. The complete mitochondrial genome of *C. himalayana* has not been determined and characterized until now. Therefore, the aim of this study was to first assemble and characterize the complete mitochondrial genome of *C. himalayana*.

The specimen (Duan-025) was collected from Zixi Mountain (25.02 N, 101.41E), which was located central Yunnan Province in China, and stored at the Herbarium of Southwest Forestry University. The total mitochondrial DNA was extracted from the muscle tissue using the Ezup Column Animal Genomic DNA Kit (Sangon, Shanghai, China). The mitogenome was sequenced using Illumina Hiseq sequencing platform (Illumina, San Diego, CA) and assembled with A5-miseq v20150522 (Coil et al. 2015). All library constructions and sequencing were performed at Sangon Bio Co., Ltd., Shanghai, China. The complete mitochondrial genome was annotated using MITOS Web server (http://mitos2.bioinf.uni-leipzig.de/index.py) (Bernt et al. 2013) and OGDRAW 1.3.1 (Greiner et al. 2019). The complete mitochondrial genome of *C. himalayana* was submitted to the NCBI database under the accession number MN624167. A maximum likelihood (ML) tree was implemented in RAxML v7.7.1 (Stamatakis 2014) under the GTR-Gamma model, and node support was calculated with 1000 bootstrap replications (Wei et al. 2016).Sequences of *Lorius chlorocercus* and *Psittacula alexandr* obtained from GenBank (MN_515396 and MK_986660) were used as outgroups to root trees following Jonsson and Fjeldsa (2006).

The complete mitochondrial genome of *C. himalayana* was found to be a circular double-stranded 16,814 bp in length. A total of 38 mitochondrial genes were identified, including 13 protein-coding genes (PCGs), 22 transfer RNA (tRNA) genes, 2 ribosomal RNA (rRNA) genes, and 1 non-coding control region (D-loop). All the 13 protein coding genes contained the same start condon TTA/TAA, except that *nad*6 and *nad*4 gene started with ATG, *nad5* gene started with TCT, *cox1* gene started with CCT. Furthermore, eleven of the PCGs used complete ATT/ATG or incomplete A(TA) stop codon, and other two types were AGG[*nad*6], ATC[*atp8*].

Among these genes, *nad*6 and 8 tRNAs (*trnE*, *trnP*, *trnS*, *trnY*, *trnC*, *trnN*, *trnA* and *trnQ*) were located on the light strand (L-strand), while all of the remaining genes were located on the heavy strand (H-strand). The overall base composition of *C. himalayana* mitogenome was 25.1% for A, 29.2% for T, 14.5% for C, 31.2% for G, A + T content is 54.3%, which is higher than G + C content of 45.7%, similar to other Psittaciformes (Barker 2014; Song et al. 2018).

The reconstructed phylogenetic tree showed that *C. himalayana* grouped with Family Troglodytidae (*Campylorhynchus brunneicapillus, C. zonatus* and *Henicorhina leucosticta*) with strong support (100% bootstrap support value) by the analyses of protein-coding genes (Figure 1). *C. himalayana* closely related with Family Troglodytidae, which is congruent with previous studies (Jonsson and Fjeldsa 2006). The complete mitochondrial genome of *C. himalayana* reported here will be useful for future population genetic studies of this species and will provide essential genome resources for the ecologically important species.

**Figure 1.**
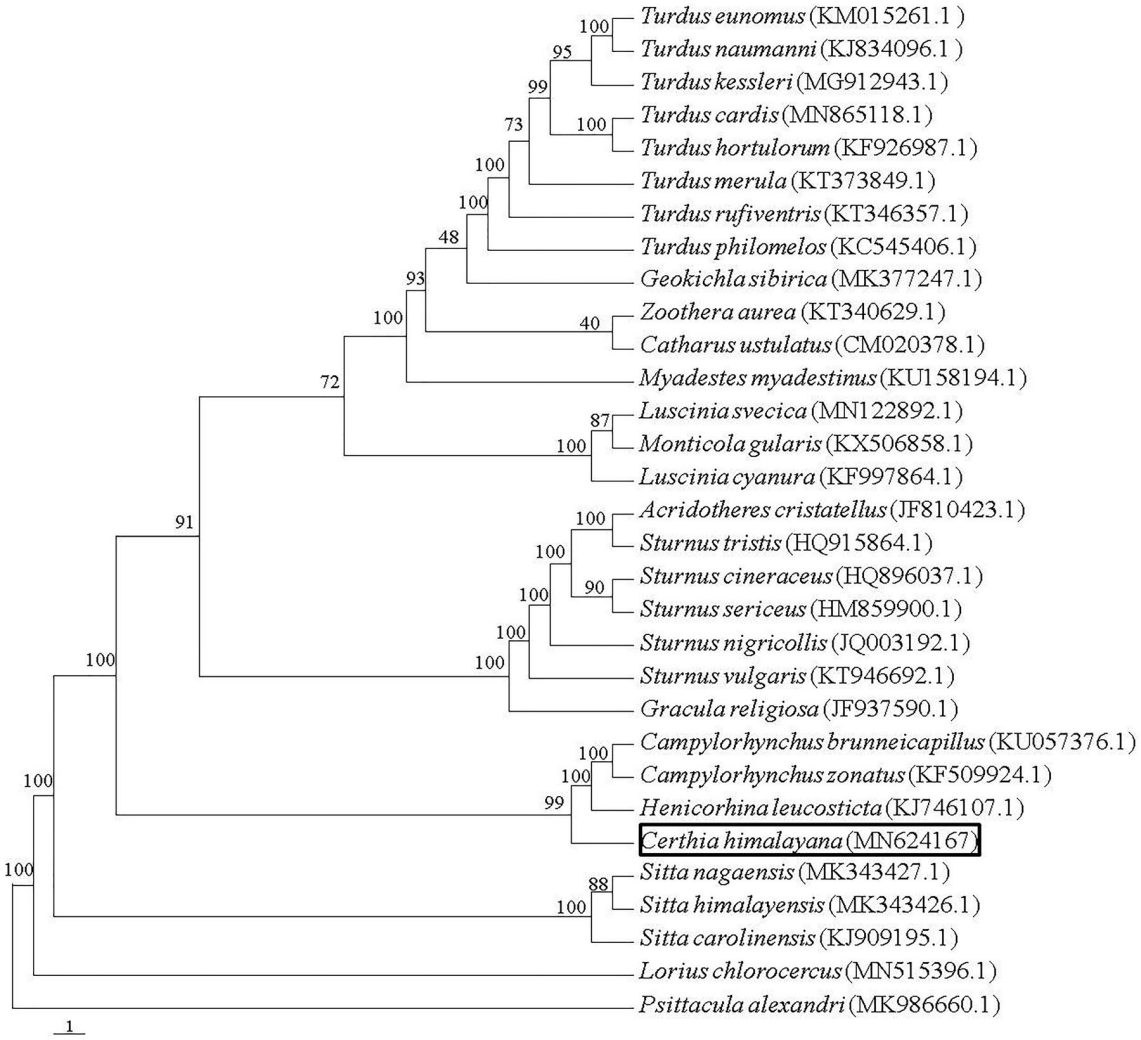
The phylogenetic tree based on combing protein-coding gene sequences of 31 speices. Numbers at node of the tree branches represent support values for maximum parsimony.

## Data Availability

The data that support the findings of this study are openly available in NCBI at https://www.ncbi.nlm.nih.gov/, reference number [MN624167], or available from the corresponding author.
